# Benznidazole-Loaded Polymeric Nanoparticles for Oral Chemotherapeutic Treatment of Chagas Disease

**DOI:** 10.3390/pharmaceutics16060800

**Published:** 2024-06-13

**Authors:** Lucas Resende Dutra Sousa, Thays Helena Chaves Duarte, Viviane Flores Xavier, Aline Coelho das Mercês, Gabriel Maia Vieira, Maximiliano Delany Martins, Cláudia Martins Carneiro, Viviane Martins Rebello dos Santos, Orlando David Henrique dos Santos, Paula Melo de Abreu Vieira

**Affiliations:** 1 Laboratório de Fitotecnologia, Programa de Pós-Graduação em Ciências Farmacêuticas, Escola de Farmácia, Universidade Federal de Ouro Preto, Campus Morro do Cruzeiro, Ouro Preto 35400-000, MG, Brazil; lucas.dutra@aluno.ufop.edu.br (L.R.D.S.); viviane.xavier@aluno.ufop.edu.br (V.F.X.); orlando@ufop.edu.br (O.D.H.d.S.); 2 Laboratório de Morfopatologia, Programa de Pós-Graduação em Ciências Biológicas, Núcleo de Pesquisas em Ciências Biológicas, Universidade Federal de Ouro Preto, Campus Morro do Cruzeiro, Ouro Preto 35400-000, MG, Brazil; thays.duarte@aluno.ufop.edu.br (T.H.C.D.); aline.merces@aluno.ufop.edu.br (A.C.d.M.); 3 Centro de Desenvolvimento da Tecnologia Nuclear, Belo Horizonte 31270-901, MG, Brazil; gabrielmaiamg@gmail.com (G.M.V.); mdm@cdtn.br (M.D.M.); 4 Laboratório de Imunopatologia, Programa de Pós-Graduação em Ciências Biológicas, Núcleo de Pesquisas em Ciências Biológicas, Universidade Federal de Ouro Preto, Campus Morro do Cruzeiro, Ouro Preto 35400-000, MG, Brazil; carneirocm@ufop.edu.br; 5 Laboratório de Produtos Naturais e de Síntese Orgânica, Programa de Pós-Graduação em Química, Instituto de Ciências Exatas e Biológicas, Universidade Federal de Ouro Preto, Campus Morro do Cruzeiro, Ouro Preto 35400-000, MG, Brazil; vivianesantos@ufop.edu.br

**Keywords:** benznidazole, Chagas disease, formulations, polymeric nanoparticles, trypanocidal activity

## Abstract

Chagas disease (CD) is a worldwide public health problem. Benznidazole (BZ) is the drug used to treat it. However, in its commercial formulation, it has significant side effects and is less effective in the chronic phase of the infection. The development of particulate systems containing BZ is therefore being promoted. The objective of this investigation was to develop polymeric nanoparticles loaded with BZ and examine their trypanocidal impact in vitro. Two formulas (BNP1 and BNP2) were produced through double emulsification and freeze drying. Subsequent to physicochemical and morphological assessment, both formulations exhibited adequate yield, average particle diameter, and zeta potential for oral administration. Cell viability was assessed in H9C2 and RAW 264.7 cells in vitro, revealing no cytotoxicity in cardiomyocytes or detrimental effects in macrophages at specific concentrations. BNP1 and BNP2 enhanced the effect of BZ within 48 h using a treatment of 3.90 μg/mL. The formulations notably improved NO reduction, particularly BNP2. The findings imply that the compositions are suitable for preclinical research, underscoring their potential as substitutes for treating CD. This study aids the quest for new BZ formulations, which are essential in light of the disregard for the treatment of CD and the unfavorable effects associated with its commercial product.

## 1. Introduction

Recognized by the World Health Organization as a neglected disease, Chagas disease (CD) represents an important public health challenge, impacting millions of patients annually in endemic countries [1,2]. It is estimated that between 6 and 7 million people are infected worldwide, the majority of them in Latin America, where the disease is endemic in 21 countries [3]. Recently, the disease has also gained importance in non-endemic areas, mainly due to the migratory movement of infected patients to countries where blood banks and organ transplants are poorly controlled [4]. In the Americas, 30 thousand new cases of CD and 10 thousand deaths are reported each year. Additionally, 75 million people are at risk of contracting the disease [3].

Chemotherapy is the only therapy available for CD, aiming to reduce the parasite count and control the infection’s progression [4,5]. At the moment, the treatment of Chagas disease is based on benznidazole (BZ) and nifurtimox, which have been the only available drugs since 1972 [5]. The prolonged therapeutic regimen with the current BZ commercial formulation, coupled with high doses, can lead to various adverse effects and frequent treatment interruptions due to this [6,7].

In many cases during the acute CD phase, BZ has successfully cured patients. However, the efficacy of chemotherapy markedly declines during the chronic phase in adult patients [8]. This issue is exacerbated by the different resistance profiles of various strains of *T. cruzi* to currently available drugs, which in turn impacts their therapeutic efficacy [8,9]. Additionally, achieving therapeutic success with BZ is challenging due to difficulties in solubility in aqueous medium and low absorption after oral administration, which are attributed to the commercially available formulation and the drug itself [10,11,12].

Developing new therapeutic strategies, such as reformulating BZ, is highly advisable. Within this framework, there is a necessity to create a new formulation of BZ to improve its plasma exposure and, therefore, its efficacy. This could potentially lead to new therapeutic opportunities for animal models and individuals with CD [13].

The development of oral formulations provides numerous pharmacotherapeutic benefits, with patient convenience being a key advantage. Oral administration is deemed a straightforward and comfortable method of drug administration, which eradicates invasive procedures such as injections and can be effortlessly integrated into the patient’s daily routine [14]. This route of administration promotes superior adherence to treatment due to its higher accessibility and cost-effectiveness [15]. Furthermore, oral administration enables the utilization of various dosage forms, thereby facilitating the customization of the formulation to the specific traits of the medication [16]. These traits encompass solubility, stability, and rate of release, thereby enhancing the drug absorption and therapeutic effectiveness [17].

Particulate systems have gained attention as a viable alternative for the development of new oral formulations. These systems comprise solid or semi-solid particles that can be orally administered and provide benefits in terms of bioavailability and stability [18].

Polymeric nanoparticles offer a number of advantages when compared to other systems. They exhibit significantly higher stability in the gastrointestinal tract compared to, for example, liposomes, which gives them the ability to protect the encapsulated drug. In addition to controlled drug release and protection, they could present prolonged blood circulation and several other adjustable features [19].

Some polymers have been used to develop polymeric nanoparticles loaded with drugs, including polyethylene glycol 4000 (PEG 4000), polycaprolactone (PCL) and poly(vinyl alcohol) (PVA) [20,21,22].

PEG 4000 is a synthetic homopolymer composed of ethylene oxide subunits. It has excellent characteristics, including water solubility, biocompatibility, and good tolerance [21,23,24]. Additionally, it allows for simple modification of the terminal group and is non-immunogenic and non-toxic [25]. The properties of PCL are exemplified by its remarkable ability to maintain drug permeability, particularly that of hydrophobic substances, such as BZ [20]. Its advantageous characteristics for drug delivery systems are numerous, making it a promising option for formulating systems at the nanoscale, especially those intended for oral administration [26,27]. Finally, PVA serves as an emulsion stabilizer and stabilizes polymeric nanoparticles not only during the emulsification stage and in solution but also in the solid state. The binding of PVA affects the hydrophilicity/hydrophobicity of a particle’s surface and its digestibility [22,28].

In this context, considering the limited oral absorption and low therapeutic efficiency of the conventional formulation of BZ in the chronic phase of CD, along with its status as the only available drug with trypanocidal action despite other available options, there is an urgent need to create new formulations that contain BZ to combat *T. cruzi*. Therefore, the promising approach of employing particulate systems with BZ gains prominence, as certain polymers such as polyethylene glycol (PEG), polycaprolactone (PCL) and polyvinyl alcohol (PVA) in oral formulations may alleviate the drug’s shortcomings and potentially bolster its effectiveness across diverse aspects. The aim of this investigation is to develop and evaluate particulate systems with BZ in vitro, for its use against *T. cruzi*.

## 2. Experimental Section

### 2.1. Development of Particulate Systems Containing Benznidazole

#### 2.1.1. Synthesis of Acetylated PEG 4000

10 g of the PEG 4000 g/mol (Sigma Aldrich, Saint Louis, MI, USA) was dissolved in 50 mL of dichloromethane (Synth, Diadema, Brazil) in a beaker, while 25 milliliters of acetic anhydride (Synth, Diadema, Brazil) were mixed with 25 mL of acetic acid (Synth, Diadema, Brazil) in another beaker. The solutions were then combined and poured into a round-bottom flask, which was positioned in an oil bath on a hot plate and connected to a reflux condenser. The oil temperature was subsequently maintained at 40 °C and the reaction was allowed to occur for 30 min. At the end of the reaction time, the solution was transferred to a separation funnel and subsequently, 100 mL of ice-cold ultrapure water was added. The acetylated PEG 4000 production yield was calculated as the ratio between the quantity of resulting particles and the total quantity of starting material. Following separation of the phases utilizing the same apparatus, the organic phase was subjected to drying at 40 °C to obtain acetylated PEG 4000 [12].

#### 2.1.2. Preparation of Particulate Systems by Double Emulsification and Freeze Drying

The polymer–drug nanoparticles were prepared by the double emulsification method with some modifications [29,30]. BZ (10 mg, 4.16% *w*/*w*) was dissolved in 1 mL of acetone and PEG 4000 g/mol (100 mg, 41.67% *w*/*w*) or acetylated PEG 4000 g/mol (100 mg, 41.67% *w*/*w*) was dissolved in 1 mL of ultrapure water. After solubilization, the solutions were mixed and the resulting solution was emulsified in a solution of PCL 80,000–90,000 g/mol (100 mg, 41.67% *w*/*w*) in dichloromethane (4 mL). This water-in-oil (W/O) emulsion was poured onto a solution of PVA 30,000–70,000 g/mol (30 mg, 12.50% *w*/*w*) in ultrapure water (30 mL) and emulsified with the aid of a mechanical stirrer (Fisatom, São Paulo, Brazil) at 3000 rpm for a minute to obtain a double emulsion (W/O/W). This emulsion was subjected to the ultradisperser (Ultra-Turrax IKA T25, Staufen, Germany) at 18 rpm × 1000 for 3 min. The organic solvent was allowed to evaporate. The formulations without BZ were prepared as previously described, but with only the polymers. The preparation procedures for the formulations and formulations without BZ were carried out three times. The emulsions were transferred into tubes and subsequently frozen in liquid nitrogen. After freezing, they were subjected to the freeze-drying process in a freeze dryer (Benchtop K, Bieleveld, Germany) for approximately 72 h to obtain solid nanoparticles. The particle production yield was calculated as the ratio between the quantity of resulting particles and the total quantity of starting material. The results are expressed as the mean of the three results ± standard deviation.

### 2.2. Physicochemical Characterization

The physicochemical properties (including particle hydrodynamic diameter, polydispersity index and zeta potential) were assessed through employment of the Zetasizer^®^ Nano Series (Malvern Instruments, Malvern, UK).

The particles’ hydrodynamic diameter and polydispersity index were assessed using dynamic light scattering based on photon correlation spectroscopy [31,32]. The samples were diluted in ultrapure water (1:1000), transferred to quartz cuvettes and evaluated at 25 ± 2 °C at a fixed angle of 90° before and after the freeze-drying process. To determine the zeta potential, samples were inserted into a container fitted with two electrodes where an electric field was applied [33]. Prior to and after freeze drying, the samples were diluted in ultrapure water at a ratio of 1:1000 and administered to a semi-disposable capillary cell via syringe. Zeta potential measurements were conducted in triplicate at a temperature of 25 ± 2 °C. The experiments were conducted thrice and the outcomes were stated as mean ± standard deviation.

### 2.3. Morphological Characterization

#### 2.3.1. Scanning Electron Microscopy (SEM)

SEM was performed on a field emission scanning electron microscope (FEG-SEM), SIGMA VP model (Carl Zeiss, Jena, Germany). For the BZ sample, a 5 mg/mL acetone solution was prepared, while for P1, P2, BNP1 and BNP2 a 5 mg/mL water suspension was prepared, magnetically stirred for 1 h, dropped onto a clean Si substrate, dried at room temperature, being finally metallized with gold and inserted into the microscope. The working distance was approximately 3 mm, at an accelerating voltage of 1 kV.

#### 2.3.2. Atomic Force Microscopy (AFM)

The atomic force characterization of the nanoparticles was carried out on a NTREGRA atomic force microscope (NT-MDT, NTEGRA II, Moscow, Russia) in air at room temperature using the semi-contact mode. Gold silicon cantilevers produced by NT-MDT, with spring constants of 2.5 to 10 N/m, resonance frequency of 115–190 kHz and with tip radius of 10 nm. The same samples prepared for SEM were used. AFM images of 5 μm^2^ and 1 μm^2^ were obtained and digitally edited for better visualization using the software Gwyddion (Version 2.59).

### 2.4. Total Benznidazole Content and Encapsulation Efficiency

#### 2.4.1. Standard Analytical Curve

In order to obtain the standard analytical curve, nine concentrations of BZ were prepared (15.00, 30.00, 45.00, 60.00, 75.00, 90.00, 105.00, 120.00 and 135.00 μg/mL) in 10.00 mL volumetric flasks using acetonitrile as solvent. The 1.50 mL aliquots of solutions were filtered using a 0.45 μM filter and transferred to vials that were subjected to HPLC (Waters Alliance 2695) coupled to a DAD detector (Waters 2996). The equipment was operated at 40 °C at 324 nm with an octadecyl-silica–C18 chromatographic column (150 × 4.6 mm × 5 μm). As the mobile phase, a mixture of water/acetonitrile (70:30, *v*/*v*) was used with an isocratic flow of 1.00 mL/min. The injection volume was 20.00 μL and the run time was 7 min (15). The straight line equation was obtained by linear regression using the area values obtained.

#### 2.4.2. Total Benznidazole Content

A sample of 4.08 mg of the formulations was dispersed in 1 mL of acetonitrile. Next, it was applied to an ultrasonic bath for 10 min, then 1 mL of ultrapure water was added to the solutions and they were subjected to another 10 min of ultrasonic bath. The solutions were collected, transferred to vials with inserts, and analyzed by HPLC in the conditions given in Section 2.4.1. The drug concentration was quantified through measuring the area values obtained utilizing the linear equation. The experiment was undertaken in triplicate and results are expressed as mean ± standard deviation.

#### 2.4.3. Encapsulation Efficiency

A 7.68 mg sample of the formulations was dispersed in 2 mL of ultrapure water. Next, 500 µL of the resulting solution was pipetted into ultrafilters with a 50 K cut-off, which were then centrifuged at 14,000 rpm for 15 min. The solution containing free BZ was collected, transferred to vials with inserts, and analyzed by HPLC in the conditions given in Section 2.4.1. The concentration of unbound medication was determined by the area values acquired using the linear equation. The experiment was conducted in triplicate, and the encapsulation efficiency (EE%) was ascertained using Equation (1). The outcomes are presented as mean ± standard deviation.

EE% = (% total BZ − % free BZ/% total BZ) × 100(1)

### 2.5. Cell Viability Using H9C2 Cardiomyocytes and RAW264.7 Macrophages

H9C2 cardiomyocytes isolated from rats (ATCC: CRL-1446) and RAW 264.7 macrophages isolated from mice (ATCC: TIB-71) were cultured in RPMI 1640 medium with 10% FBS added until 80 to 100% confluence was reached. Afterwards, 5 × 10^5^ cells were distributed in 96-well plates and incubated at 37 °C with 5% CO_2_ for 24 h. The cells were treated with the formulations (BNP1 and BNP2) at six concentrations (125.00, 62.50, 31.25, 15.62, 7.81 and 3.90 µg/mL). Free BZ was used as the treatment at the same concentrations. The formulations without BZ (P1 and P2) were evaluated at the polymer concentrations present in the BNP1 and BNP2 formulations (2875.00, 1437.50, 718.75, 359.37, 179.69 and 89.84 µg/mL). All samples were prepared in RPMI + 5% DMSO. The cells were also treated with 5% DMSO to prove that this solvent, at this concentration, does not interfere with cell viability. Cell viability was assessed using MTT 24 and 48 h after incubation with treatments [34]. Absorbance was read on a microplate reader (Infinite® 200 PRO, TECAN, Männedorf, Switzerland) at 490 nm and percentage cell viability was determined using GraphPad Prism 8.0.1 software. The assay was performed in triplicate. The results were expressed as the mean of triplicates ± standard deviation.

### 2.6. In Vitro Trypanocidal Activity against Amastigote Forms of the Y Strain of T. cruzi

H9C2 cells were cultured in RPMI 1640 medium at a density of 5 × 10^5^ in 24-well plates containing 13 mm coverslips. The plates were then incubated in an oven at 37 °C with 5% CO_2_ for 24 h. After this period, metacyclic trypomastigote forms of the Y strain of *T. cruzi* were added at a density of 1 × 10^6^. Four hours after infection, treatments with BNP1, BNP2 and BZ (positive control) were added at four concentrations (31.25, 15.62, 7.81 and 3.90 µg/mL). The concentrations of polymers in the BNP1 and BNP2 formulations (718.75, 359.37, 179.69 and 89.84 µg/mL) were evaluated for the P1 and P2 formulations without BZ. All treatments were prepared with 5% DMSO, which was also evaluated as a negative control. Untreated infected cells and uninfected and untreated cells were also incubated to compare their morphology with infected cells. The plates were incubated for 24 and 48 h. After the treatment period, the supernatant was discarded, and the coverslips were stained with quick panoptic. The coverslips were then dried and affixed to previously identified matte tip slides using Entellan^TM^ (Merck, Darmstadt, Germany). The slides were analyzed under a microscope (Leica-DM 25,000, Wetzlar, Germany) at 100x magnification, using IR 1.515 immersion oil. The number of infected cells per 100 was counted in triplicate, along with the total number of amastigotes in 100 cells. The results were analyzed using GraphPad Prism 8.0.1 (GraphPad Software, San Diego, CA, USA) and expressed as the mean ± standard deviation. Photomicrographs were captured using Leica Application Suite version 4.10.0 software to represent the results.

### 2.7. Nitric Oxide Measurement in RAW 264.7 Macrophage Supernatants

RAW 264.7 macrophages were cultured in RPMI 1640 medium, including 10% FBS, until they reached 80 to 100% confluence. Afterwards, we transferred 5 × 10^5^ cells to 96-well plates and incubated them at 37 °C with 5% CO_2_ for 24 h. Following this, the cells were either stimulated or not with LPS (10 μg/mL) and IFN-γ (40 ng/mL), and then treated with BNP1 and BNP2 at four concentrations (31.25, 15.62, 7.81 and 3.90 µg/mL). BZ was utilized as a positive control and treated with the same concentrations. The polymer concentrations present in the BNP1 and BNP2 formulations (718.75, 359.37, 179.69 and 89.84 µg/mL) were assessed for P1 and P2 formulations without BZ. All samples were prepared in RPMI + 5% DMSO. The Griess reaction was used to measure NO, with supernatant collected following incubation with the treatments after 24 and 48 h [35]. The absorbance was measured using a microplate reader (Infinite® 200 PRO, TECAN, Männedorf, Switzerland) at 570 nm, and the nitrite concentration was calculated using the sodium nitrite solutions’ obtained analytical curve. The process was repeated thrice, and GraphPad Prism 8.0.1 software was used to evaluate and display the results as the mean of the triplicates ± standard deviation.

### 2.8. Statistical Analyses

Statistical analyses were conducted using GraphPad Prism 8.0.1 (GraphPad Software, San Diego, CA, USA). The Shapiro–Wilk test was employed to determine the normal distribution of the particle diameter, PDI, zeta potential, cell viability, in vitro trypanocidal activity, and NO levels. The particle diameter, PDI and zeta potential results were analyzed using the T test, assessing variations between the two formulations before and after freeze drying, and the differences in each formulation before and after the freeze-drying process. The results of cell viability and in vitro trypanocidal activity were subjected to a one-way ANOVA test with Dunnett’s multiple comparisons post test to explore contrasts between the formulations containing and not containing BZ. A one-way ANOVA test was also run on the NO results to examine distinctions between the sample groups concerning both stimulated and untreated control groups, with significance deemed at *p* < 0.05.

## 3. Results and Discussion

### 3.1. Obtainment and Percentage Yield of Acetylated PEG 4000 and of the Formulations

**Figure 1:**
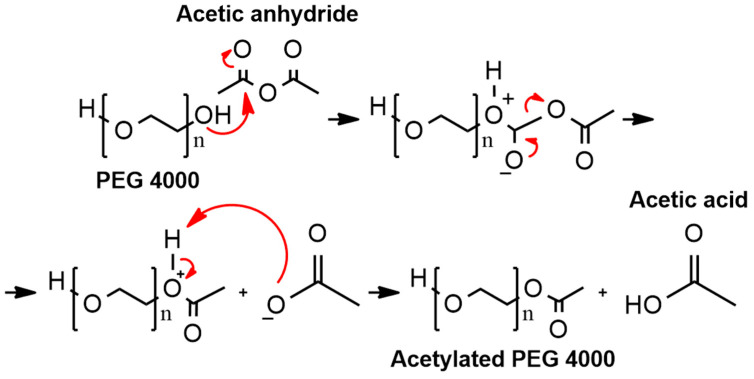
Partial chemical reaction to obtain acetylated PEG 4000.

Chemical modifications are processes that can confer several advantages in terms of drug loading [36]. In this context, acetylation was performed in PEG 4000 and characterized by NMR, in accordance with our previous publication [14]. During the acetylation process, the hydroxyl group of PEG 4000 attacks the carbonyl carbon of acetic anhydride at one or both ends, resulting in the partial or complete modification of the functional group at these locations (Figure 1).

PEG 4000 was employed for acetylation, given that simple acetylated PEG chains have been demonstrated to function as highly efficacious drug carriers. To illustrate, the simple acetylation of the PEG terminus results in the aggregation of PEG chains into spherical nanoparticles in water at room temperature and very low concentrations. This aggregation occurs as a result of the alteration of the local conformation of the O–CH_2_–CH_2_–O segments of the PEG chains, brought about by the introduction of the acyl group. This disrupts the previously established hydrogen bond mode between the O–CH_2_–CH_2_–O groups and the water molecules. As a result, the modified PEG nanoparticles are excellent drug carriers [36]. Furthermore, an increase in the number of organic functional groups could enhance the interaction with the organic chain of BZ and simultaneously promote an increase in the solubility of this drug in water, which could improve its efficacy.

Following the acquisition of acetylated PEG 4000, particulate systems loaded with BZ were developed with the objective of creating a product that would overcome the low oral absorption of BZ. The particulate systems (BNP1 and BNP2) were prepared using the double emulsification method followed by freeze drying to physically stabilize the suspension. In addition, some authors suggest that a second step in the emulsification process can optimize the technique for preparing polymeric nanoparticles [37,38]. Furthermore, previous studies have shown that the use of PVA can also enhance this process, with the degree of improvement being directly related to its concentration [39]. This process yielded more than 90% (Table 1).

### 3.2. Particle Diameter and Polydispersity Index

**Figure 2:**
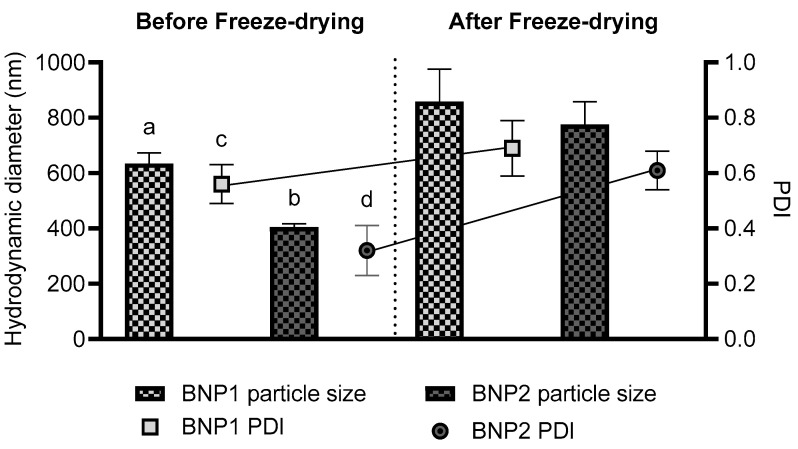
Average particle diameter and PDI of particulate systems before and after freeze drying. Results expressed as the mean of triplicates ± standard deviation. a: significant difference in relation to the size of BNP2 before freeze drying and in relation to the size of BNP1 after freeze drying; b: significant difference in relation to the size of BNP2 after freeze drying; c: significant difference in relation to the PDI of BNP2 before freeze drying; d: significant difference in relation to the PDI of BNP2 after freeze drying.

Polymeric particles loaded with drugs with sizes smaller than 1000 nm are considered nanoparticles, while those larger than this value are classified as microparticles [40]. In addition, the evaluation of the PDI is usually carried out together with the mean particle diameter, as this parameter provides information on the homogeneity of the distribution of this mean size. Polydispersity indices below 0.3 represent monodisperse systems, up to 0.7 indicate moderate polydispersity, while those above 0.7 indicate a broad size distribution [41,42,43,44]. In this study, the average particle diameter and polydispersity index were assessed before and after the freeze-drying process (Figure 2).

Both formulations showed a nanometric particle size with moderate distribution, as evidenced by their size below 1000 nm and PDI up to 0.7, both before and after freeze drying. The PDI between 0.3 and 0.7 found for the formulations may present a challenge, particularly if the current formulations were developed for parenteral use, for instance [45]. However, as these are formulations intended for oral use, the moderate variation between sizes is acceptable, primarily because the objective is to enhance the solubility of BZ in water media and consequently boost bioavailability.

It is important to highlight that in certain instances, an enhancement in both the average diameter and PDI was observed subsequent to the freeze-drying process, particularly in the absence of cryoprotectants throughout this process [46]. Additionally, researchers observed an increase in average particle diameter and differences in PDI based on the proportions of polymer used, as well as variations in the molecular weights of the polymer. Dos Santos-Silva et al. (2017) observed changes in average size and PDI when BZ was added to the systems under study [47]. A study conducted by Streck et al. (2016) involving nanoemulsions supported the previous findings, demonstrating a change in particle size following the addition of BZ [48]. In addition to the changes that may be related to the polymer–drug relationship, the lyophilization process itself can lead to changes in the mean diameter and PDI after powder reconstitution due to the stress caused by freezing and sublimation inherent to the technique [40,47,49,50,51]. A study using a double emulsification technique followed by lyophilization, similar to the present study, to produce triamcinolone-loaded PLA and PVA microparticles showed an average particle size of 2 μm [22]. Another study showed particle sizes between 94 and 175 nm of PEG and PCL loaded with potentially active compounds obtained by the double emulsion evaporation process [52]. These results confirm that the differences in particle size observed are due to the use of different techniques, polymers, active compounds and the absence of cryoprotectants during lyophilization.

### 3.3. Zeta Potential

Zeta potential is a measurement of the electrical charge on the surface of particles in various formulations. In order to maintain the stability of formulations, zeta potential values above 25 mV in modulus are generally considered ideal. This is due to the fact that under these conditions, repulsion forces between particles predominate, preventing their aggregation [40,53].

The zeta potential of BNP1 before freeze drying was markedly lower than that of BNP2. Following freeze drying, there were no significant differences in the zeta potential between both BNP1 and BNP2 (Table 2).

A possible good stability was observed after freeze drying for both BNP1 and BNP2. In general, the formulations presented zeta potential values close to those indicated, and the PEGs present in the constitution also provided stabilization through a steric effect. This effect occurs due to the bulky surface groups present in these polymers, which minimize the probability of particle aggregation [54,55].

Apparently, PEG and PCL nanoparticles impart a negative charge to newly developed systems [52,56]. In one study, the zeta potential of PCL-PEG-PCL triblock copolymers loaded or unloaded with curcumin was −22.73 mV and −29.23 mV [56]. Another study using PEG and PCL nanoparticles showed nanoparticles with zeta potentials of −20.2 mV and −22.3 mV [52]. Both showed results that corroborate our findings.

### 3.4. Scanning Electron Microscopy (SEM)

**Figure 3:**
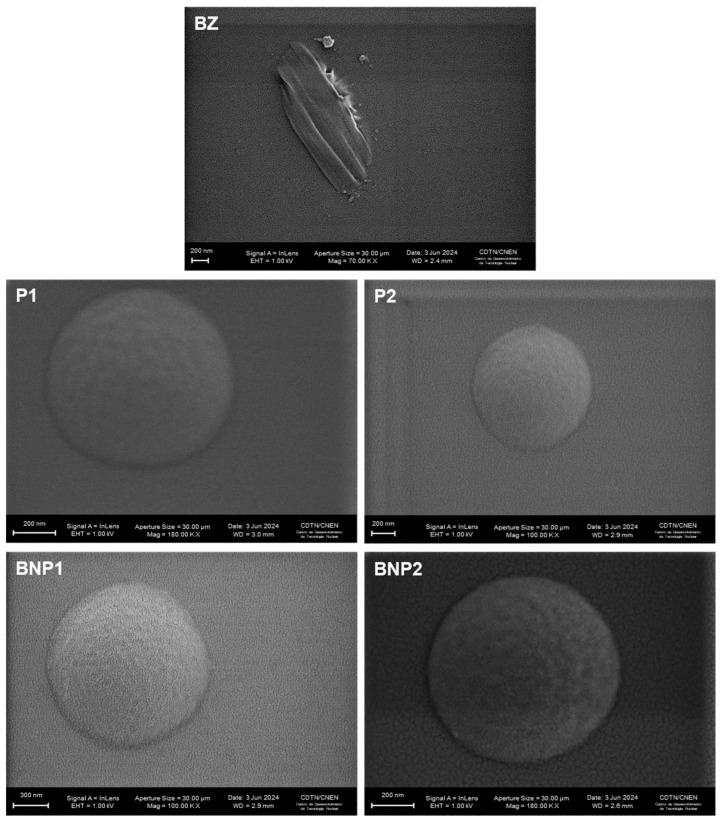
Morphology of the BZ and of the nanospheres (P1, P2, BNP1 and BNP2) obtained by scanning electron microscopy. The white scale bars represent 200 nm or 300 nm.

Figure 3 displays photomicrographs of BZ, formulations without BZ and with BZ.

The drug (BZ) exhibited uneven surfaces, resembling rods, as seen. Other researchers have also observed photomicrographs of the same drug [57]. The formulations P1, P2, BNP1 and BNP2 were spherical and had nanometer-scale sizes, which is consistent with the average hydrodynamic diameter and PDI obtained by the zetasizer.

Looking at the literature, the study by Leonardi et al. (2013) showed BZ particles in morphologically similar cyclodextrin complexes by SEM [58]. Furthermore, PLA-PVA microspheres loaded with triamcinolone presented particles similar to those in the present work, apparently with a slightly smoother surface, which may be due to differences in composition [22].

### 3.5. Atomic Force Microscopy (AFM)

**Figure 4:**
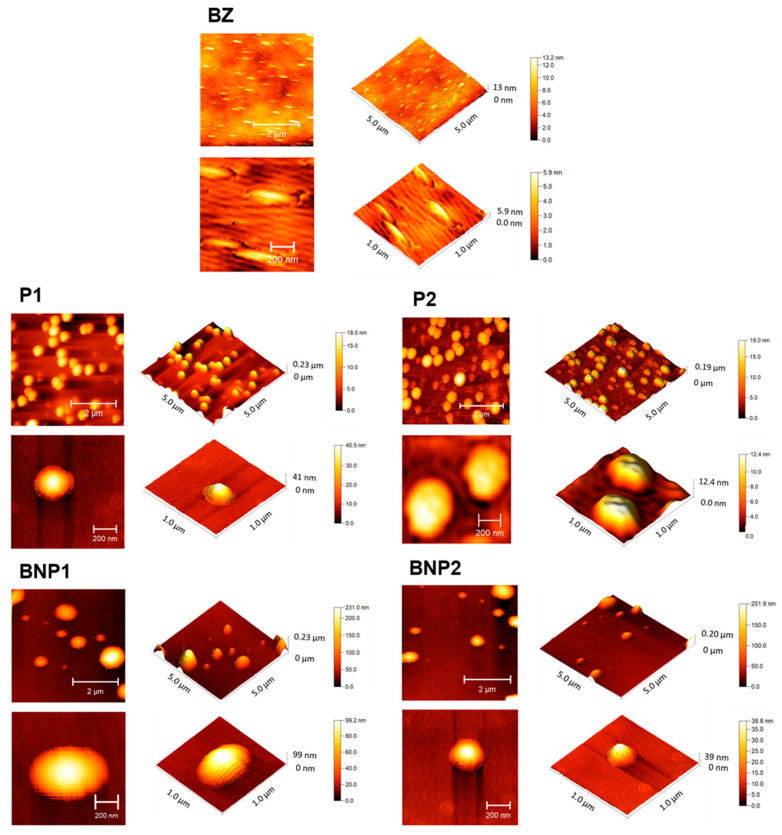
Morphology of the BZ and of the nanospheres (P1, P2, BNP1 and BNP2) obtained by atomic force microscopy. The white scale bars represent 200 nm or 2 μm.

AFM analysis (Figure 4) revealed that BZ showed a rod shape. On the other hand, P1, P2, BNP1 and BNP2 presented spherical or oval shapes, which corroborates the SEM results. In terms of particle size, the AFM images revealed smaller nanoparticles (200–300 nm) when compared to the DLS results, which can be explained by the dehydration of the nanoparticles submitted for AFM imaging [59].

### 3.6. Total BZ Content and Encapsulation Efficiency

**Figure 5:**
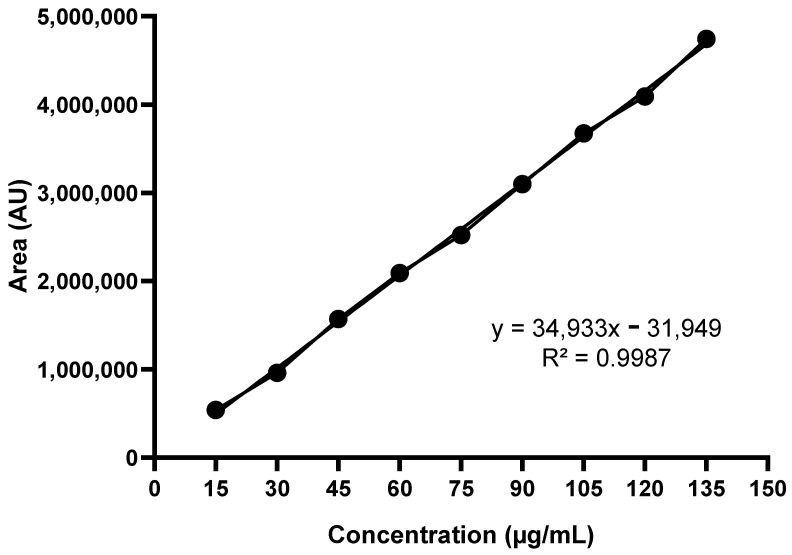
Standard analytical curve prepared with BZ.

When developing new formulations, it is important to measure both the total percentage and the percentage of the active ingredient that is encapsulated. For this evaluation, we employed the standard analytical curve prepared with BZ, for which the chromatographic signal areas were obtained at 324 nm (Figure 5, Table 3).

Both BNP1 and BNP2 had a calculated total content above 90%. This was expected due to residual amounts of drug or polymer that may be retained in the preparation glassware during formulation. In both cases, values close to 70% were found for EE. This finding corroborates the study by Abriata et al. (2017), which showed an EE between 36.44% and 94.10% for BZ-loaded polymeric nanoparticles obtained by nanoprecipitation [60]. Drug loss is expected in both cases, as hydrophobic drugs such as BZ easily escape from the polymer wall during solvent evaporation, making the EE dependent on the polymer–drug interaction [61,62,63]. In a previous study conducted by our research group, EE (%) values between approximately 30 and 40% were obtained for similar formulations [12]. In the present study, one of the objectives was to optimize both the technique for obtaining particulate systems and to evaluate the influence of the addition of PCL on the formulations, including on EE (%). It was observed that with the changes made, the EE (%) increased.

### 3.7. Cell Viability Assay

Cell viability studies are important in determining the potential toxic effects of the systems developed and establishing safe doses for their intended application [64,65]. The reference used in this project was ISO 10993-5 from 2009, which defines cytotoxicity as viability below 70% [66].

For this evaluation, H9C2 cardiomyocytes were cultured [67]. This cardiac cell line was isolated from rats and is commonly used as an in vitro model to study the biology and physiology of cardiomyocytes [67,68]. It is also used to investigate processes related to heart disease and to evaluate the effectiveness of new therapies [65,69]. Cell viability was also evaluated in RAW 264.7 macrophages, which were derived from mice [70]. Macrophages are cells of the immune system that defend the body against pathogens and regulate the inflammatory response. They are often used to assess the immune response [71].

**Figure 6:**
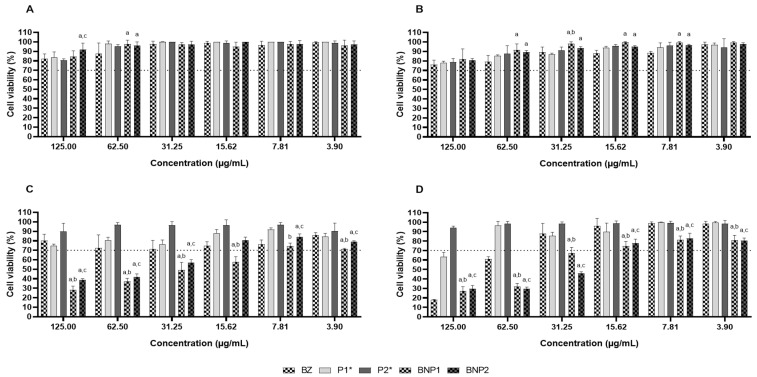
Cell viability of H9C2 cardiomyocytes treated for 24 h (**A**) and 48 h (**B**) and of RAW 264.7 macrophages treated for 24 h (**C**) and 48 h (**D**) with BZ, P1, P2, BNP1 and BNP2. DMSO 5%: negative control; *: concentration corresponding to the concentration of polymers used in BNP1 and BNP2; a: significant difference in relation to BZ; b: significant difference in relation to P1; c: significant difference in relation to P2. Results expressed as the mean of triplicates ± standard deviation.

No cytotoxicity was observed in any of the samples against H9C2 cardiomyocytes at all evaluated concentrations after 24 and 48 h. Notably, at 24 h, the BNP1 formulation showed lower cytotoxicity than BZ at 62.50 μg/mL and also than BNP2 at 125.00 and 62.50 μg/mL. Interestingly, BNP2 was less cytotoxic than P2 at 125.00 μg/mL (Figure 6A). After 48 h, both formulations were less cytotoxic than free BZ at 62.00, 31.25, 15.62 and 7.81 μg/mL. Moreover, the BNP1 formulation displayed less cytotoxicity than P1 at 31.25 μg/mL (Figure 6B). From the evaluation conducted on RAW 264.7 macrophages over a period of 24 h, it can be inferred that the samples BZ, P1, and P2 did not exhibit cytotoxicity at any of the tested concentrations. The BNP1 formulation was found to affect cell viability only at concentrations lower than 7.81 μg/mL, while BNP2 was not cytotoxic at concentrations below 15.62 μg/mL (Figure 6C). However, the evaluation conducted over 48 h inferred that P2 was the only sample that did not cause any cytotoxicity at any of the concentrations tested, which contrasts with the cell viability assessment results observed in the 24 h evaluation period. At the highest concentration, sample P1 displayed cytotoxic effects whereas at lower concentrations, it had no cytotoxic effect. BZ demonstrated non-cytotoxic activity only at concentrations lower than 31.25 μg/mL. Neither BNP1 nor BNP2 formulations resulted in any impairment to cell viability at concentrations lower than 15.62 μg/mL. Interestingly, when BNP1 was purchased as BZ, increased cytotoxicity was observed at 125.00, 62.00, 31.25, 15.62, and 3.90 μg/mL. Moreover, at concentrations of 125.00, 62.00, 31.25, and 3.90 μg/mL, BNP2 exhibited greater cytotoxicity compared to BZ, whereas at 15.62 and 7.81 μg/mL, it was less cytotoxic than BZ. BNP1 exhibited higher cytotoxicity than P1 at all tested concentrations, while BNP2 showed similar results compared to P2, which also exhibited higher cytotoxicity at all seven evaluated concentrations. Furthermore, we observed an increase in cytotoxicity for both formulations within 48 h, in comparison to both BZ and the respective formulations without BZ at all concentrations tested (Figure 6D).

Nanostructured systems are often recognized as foreign bodies by immune system cells upon administration, which can lead to toxicity as the immune cells attempt to eliminate them [72]. This can result in the rapid release of the carried drug and lead to toxicity [73]. The internalization of particles by phagocytic cells of the mononuclear phagocytic system is one of the main challenges when delivering assets based on particulate carriers [74].

The properties of particulate systems, such as hydrophilicity, hydrophobicity, size, surface charge, and steric effects, can affect recognition by macrophages [75,76]. It is generally believed that particles with a predominantly hydrophobic character, larger than 200 nm, with some type of charge and no functionalization are phagocytosed more efficiently [76,77,78]. It is believed that the decrease in viability in this cell type may have been caused by the hydrophobic nature of PCL, despite the hydrophilic character promoted by PEGs. This could be attributed to the fact that the average particle size found was greater than 200 nm and the surface charge of the formulations. De Moraes et al. (2018) conducted a study that demonstrated a decrease in macrophage viability when treated with nanostructured drug-containing particles, supporting our findings [79].

It is important to note that the in vitro cytotoxicity assay using RAW 264.7 macrophages involved direct exposure of the particulate systems to the cells. However, it should be noted that the formulations were designed for oral administration, which offers several advantages over other routes of administration [14,16]. By this route, the nanoparticles will probably be decomposed before absorption, reducing, or eliminating their recognition by macrophages, as BZ would be absorbed in its free form [80,81]. It is postulated that the potential benefits of these formulations may not be directly related to the intact absorption of nanoparticles loaded with BZ. Rather, it is hypothesized that the formulations may facilitate the absorption and bioavailability of the drug, as well as promote adhesion in the stomach for drug release, which may contribute to a reduction in the toxic effects of BZ [82,83].

### 3.8. In Vitro Trypanocidal Assay

The study evaluated the effectiveness of BZ-containing formulations against amastigote forms of the Y strain of *T. cruzi*. This protocol was chosen as it is commonly found in patients with chronic CD and is important in screening new treatments. Additionally, it was important to assess the effectiveness of BNP1 and BNP2 in a strain that is partially resistant to BZ treatment [9].

**Figure 7:**
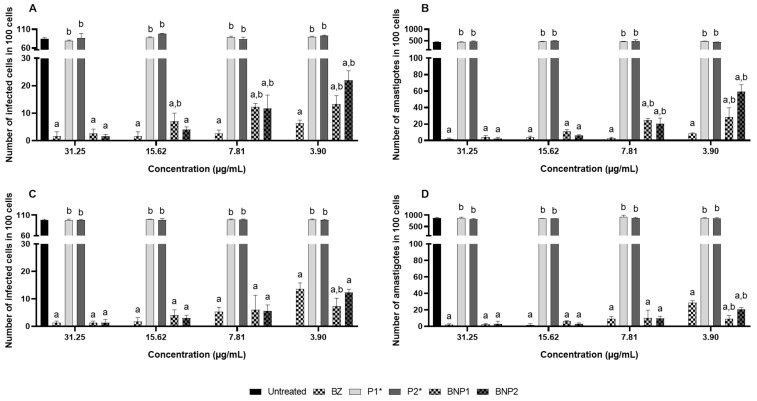
Number of infected cells (**A**) and number of amastigotes in 100 cells treated for 24 h (**B**). Number of infected cells (**C**) and number of amastigotes in 100 cells treated for 48 h (**D**). DMSO 5%: negative control; *: concentration corresponding to the concentration of polymers used in BNP1 and BNP2; a: significant difference in relation to untreated; b: significant difference in relation to BZ. Results expressed as the mean of triplicates ± standard deviation.

**Figure 8:**
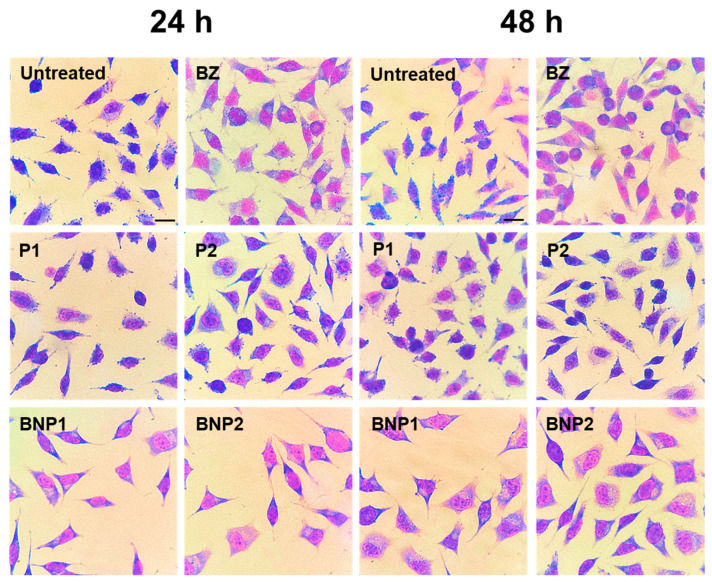
Representative photomicrographs of the treatments with the samples observed 24 h and 48 h post-treatment. Untreated, P1 and P2: intense or moderate parasitic load; BZ: low parasitic load; BNP1 and BNP2: low parasitic load. The black scale bars represent 10 μm.

The results of the anti-*T. cruzi* formulations at 24 and 48 h after treatment exhibited some variations. The number of infected cells was higher in the three lowest concentrations (15.62, 7.81 and 3.90 μg/mL) of BNP1 and in the two lowest concentrations (7.81 and 3.90 μg/mL) of BNP2 compared to treatments with BZ. Additionally, the number of amastigotes was higher in the two lowest concentrations of BNP1 and BNP2 (Figure 7A,B). However, a contrasting pattern was observed in treatments lasting 48 h. Overall, at higher concentrations, both free BZ and BNP1 and BNP2 maintained similar efficacy. Nevertheless, the BNP1 formulation was able to reduce the number of infected cells compared to BZ at the lowest concentration, just as BNP1 and BNP2 were able to reduce the number of amastigotes at 3.90 μg/mL (Figure 7C,D and Figure 8).

The lower treatment activity observed with the 24-treatment formulations compared to BZ may be attributed to the possibility of the slow release of drugs from formulations containing PEG and PCL polymeric carriers [52,84]. It is possible that BZ was released in concentrations insufficient to promote a reduction like that observed in treatments with free BZ at the concentrations mentioned. At 48 h after the treatments, in addition to the effectiveness advantages of the formulations compared to free BZ, it is noteworthy that BNP1 and BNP2 were less cytotoxic to H9C2 cardiomyocytes at concentrations of 15.62 and 7.81 μg/mL, as demonstrated previously. This suggests that their application at such concentrations could be advantageous.

When comparing these results with other anti-*T. cruzi* in vitro studies in the literature, similar results were observed. For instance, the work that used BZ loaded into polymeric particles obtained by solvent evaporation showed that the activity of the formulations against trypomastigote forms of the Tulahuen strain of *T. cruzi* was maintained even when applied in lower doses than free BZ [12]. Another study used calcium carbonate nanoparticles loaded with BZ and demonstrated a reduction in the infected cells and amastigotes of the Y strain of *T. cruzi*, similar to that of free BZ. Interestingly, lower doses of nanoparticles were required compared to free BZ to achieve these results [85].

Furthermore, polymeric carriers offer several advantages, including the potential to reduce adverse effects, increase drug stability, and improve the solubility and effects of the drug [86,87]. In this context, studies evaluating BZ dissolution in new formulations and in in vivo activity have demonstrated an increase in both dissolution and activity [84,88,89,90]. These findings suggest that the formulations presented in this work hold promise. Therefore, the use of these systems may be more advantageous compared to commercial formulation.

### 3.9. Nitric Oxide Measurement

During the development of CD, nitric oxide (NO) may be produced as a result of cellular destruction caused by *T. cruzi*, immune-mediated reactions, and damage to mitochondria [91]. Therefore, measuring the indirect levels of NO is an important indicator of potential oxidative imbalance.

**Figure 9:**
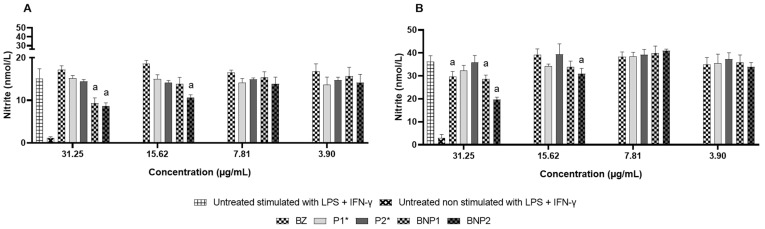
NO measurement in the supernatant of RAW 264.7 macrophages treated for 24 h (**A**) and 48 h (**B**) with BZ, P1, P2, BNP1 and BNP2. DMSO 5%: negative control; *: concentration corresponding to the concentration of polymers used in BNP1 and BNP2; a: significant difference in relation to the untreated stimulated with LPS + IFN- γ control. Results expressed as the mean of triplicates ± standard deviation.

A decrease in NO levels of 31.25 μg/mL occurred within 24 h due to the BNP1 formulation. The BNP2 formulation managed to reduce the two most concentrated samples (31.25 and 15.62 μg/mL). No differences were found in the other tested samples (Figure 9A). After 48 h, BZ and the BNP1 formulation reduced the NO concentration by 31.25 μg/mL. The reduction in NO levels was sustained by the BNP2 formulation at the two most extended concentrations (Figure 9B).

Based on the concentrations at which cell viability remained above 70%, it could be suggested that both BZ and BNP2 have the potential to reduce the pro-inflammatory response caused by NO at viable concentrations, indicating the possibility of reducing oxidative imbalance. The results obtained corroborate those of Revelli et al. (1999), who showed a reduction in NO production by RAW 264.7 macrophages stimulated and not stimulated with LPS and/or IFN-γ for 24 h after treatment with BZ [92].

During the acute phase of CD, there is an intense inflammatory process in the vertebrate host’s body. Studies indicate that, in *T. cruzi* infection, there is an increase in NO production, which aims to contain the parasite [93,94]. However, excessive amounts of NO can have undesirable effects due to its pro-inflammatory effect, even though it is also responsible for the destruction of circulating forms of the parasite [95]. Excess NO has been shown to have an oxidative effect that impairs the control of CD pathogenesis. This effect can cause damage to cells in the spleen, heart, and even neurons in the myenteric plexus. The latter may be related to the development of the digestive form of CD [96,97]. Moreover, cardiomyocytes (which are important targets of *T. cruzi*) produce high amounts of cytokines, chemokines, metalloproteinases, and NO-synthetase in response to the parasite. This leads to inflammation and subsequent cardiac remodeling [98,99]. Additionally, studies indicate that Chagasic myocarditis results from an imbalance of oxidative stress caused by lesions in cardiac cells, which reinforces the damage caused by the excessive pro-inflammatory effect of NO [91].

Therefore, treatments that reduce oxidative imbalance are essential for reducing the development of more severe forms of CD [91]. In this scenario, the use of particulate systems containing BZ would be beneficial in CD treatment due to the reduction in NO production compared to the stimulated and untreated control. Enhancing the effect of BZ would avoid the exacerbated pro-inflammatory profile triggered in the presence of *T. cruzi*. Thus, the administration of BNP2 could potentially aid in the progression or stabilization of the clinical condition of patients with CD. This is due to the known reduction in production of other pro-inflammatory mediators following the action of BNP2 [100].

## 4. Conclusions

In summary, the characterization revealed physicochemical and morphological characteristics that are consistent with the intended route of administration, possibly due to the use of different polymers. Furthermore, the absence of cytotoxicity, especially at lower concentrations, suggests the safety of polymeric nanoparticles. Furthermore, they were able to increase the trypanocidal effect of BZ over time, in addition to promoting NO reduction, which is promising for clinical practice. Thus, polymeric nanoparticles loaded with BZ emerge as promising alternatives for the oral chemotherapy of CD. However, further studies are necessary to optimize the formulation and to evaluate the dissolution and controlled release. Following such evaluations, further preclinical studies are necessary, with a particular focus on pharmacokinetics, safety and efficacy.

## Figures and Tables

**Table 1:** Yield (%) of the acetylated PEG 4000 and of the formulations.

Sample	Yield (%)
Acetylated PEG 4000	94.6
BNP1	96.57 ± 0.35
BNP2	98.17 ± 0.48

**Table 2:** Zeta potential before and after freeze drying particulate systems. Results expressed as the mean of triplicates ± standard deviation.

Formulation	Zeta Potential (mV)	
	Pre-Freeze Drying	Post-Freeze Drying
BNP1	−25.0 ± 4.12 ^a^	−21.4 ± 3.30
BNP2	−15.1 ± 1.69	−21.4 ± 4.62

^a^: significant difference in relation to the zeta potential of BNP2 before freeze drying.

**Table 3:** Total BZ content (%) and encapsulation efficiency (%). Results expressed as the mean of triplicates ± standard deviation.

Formulation	Total BZ (%)	EE (%)
BNP1	91.83 ± 0.14	69.11 ± 3.46
BNP2	95.08 ± 1.04	70.83 ± 1.05

## Data Availability

All data generated or analyzed during this study are included in this published article.

## Abbreviations

FBS	Fetal bovine serum
BZ	Benznidazole
BNP1	PEG 4000, PCL and PVA nanospheres-loaded with BZ
BNP2	Acetylated PEG 4000, PCL and PVA nanospheres-loaded with BZ
CD	Chagas disease
DMSO	Dimethyl sulfoxide
EE	Encapsulation efficiency
HPLC	High performance liquid chromatography
IFN-γ	Interferon-gamma
ISO	International Organization for Standardization
LPS	Lipopolysaccharide
MTT	3-(4,5-Dimethyl-2-thiazolyl)-2,5-diphenyl-2H-tetrazolium bromide
NMR	Nuclear magnetic resonance
NO	Nitric oxide
P1	PEG 4000, PCL and PVA nanospheres
P2	Acetylated PEG 4000, PCL and PVA nanospheres
PDI	Polydispersity index
PCL	Polycaprolactone
PEG	Polyethylene glycol
PVA	Polyvinyl alcohol
RPMI-1640	Roswell Park Memorial Institute 1640 medium
SEM	Scanning electron microscopy
AFM	Atomic force microscopy
